# Single-cell profiling of the human decidual immune microenvironment in patients with recurrent pregnancy loss

**DOI:** 10.1038/s41421-020-00236-z

**Published:** 2021-01-04

**Authors:** Chuang Guo, Pengfei Cai, Liying Jin, Qing Sha, Qiaoni Yu, Wen Zhang, Chen Jiang, Qian Liu, Dandan Zong, Kun Li, Jingwen Fang, Fangting Lu, Yanshi Wang, Daojing Li, Jun Lin, Lu Li, Zhutian Zeng, Xianhong Tong, Haiming Wei, Kun Qu

**Affiliations:** 1 Department of Oncology, The First Affiliated Hospital of USTC, Division of Molecular Medicine, Hefei National Laboratory for Physical Sciences at Microscale, Division of Life Sciences and Medicine, University of Science and Technology of China, Hefei, Anhui 230027 China; 2 HanGene Biotech, Xiaoshan Innovation Polis, Hangzhou, Zhejiang 311200 China; 3 The First Affiliated Hospital of USTC, University of Science and Technology of China, Hefei, Anhui 230021 China; 4 CAS Center for Excellence in Molecular Cell Sciences, The CAS Key Laboratory of Innate Immunity and Chronic Disease, University of Science and Technology of China, Hefei, Anhui 230027 China; 5 School of Data Science, University of Science and Technology of China, Hefei, Anhui 230027 China

**Keywords:** Immunology, Gene expression profiling

## Abstract

Maintaining homeostasis of the decidual immune microenvironment at the maternal–fetal interface is essential for placentation and reproductive success. Although distinct decidual immune cell subpopulations have been identified under normal conditions, systematic understanding of the spectrum and heterogeneity of leukocytes under recurrent miscarriage in human deciduas remains unclear. To address this, we profiled the respective transcriptomes of 18,646 primary human decidual immune cells isolated from patients with recurrent pregnancy loss (RPL) and healthy controls at single-cell resolution. We discovered dramatic differential distributions of immune cell subsets in RPL patients compared with the normal decidual immune microenvironment. Furthermore, we found a subset of decidual natural killer (NK) cells that support embryo growth were diminished in proportion due to abnormal NK cell development in RPL patients. We also elucidated the altered cellular interactions between the decidual immune cell subsets in the microenvironment and those of the immune cells with stromal cells and extravillous trophoblast under disease state. These results provided deeper insights into the RPL decidual immune microenvironment disorder that are potentially applicable to improve the diagnosis and therapeutics of this disease.

## Introduction

Recurrent pregnancy loss (RPL), defined as loss of two or more consecutive pregnancies, affects up to 5% of women trying to conceive^1,2^. Known causes of RPL include genetic abnormalities, endocrine disorders, uterine malformations, as well as other influencing factors such as thrombophilia and maternal infections^3^. The etiology of RPL remains unknown in about 50% of cases. Studies have shown that abnormalities of the decidual immune microenvironment might associate with the pathogenesis of RPL^4^. However, the underlying mechanisms through which dysregulation of the decidual immune microenvironment causes RPL remain unclear.

The decidual immune cells at the maternal–fetal interface are mainly composed of natural killer (NK) cells, macrophages, T cells, and a variety of minority cell types (e.g., dendritic cells, NKT cells, etc.)^5^, and their proportion changes with gestational age^6^. Decidual NK (dNK) cells represent the largest population, comprising about 50–70% of the maternal immune cells during the first trimester of pregnancy^7^. Studies have shown that dNK cells exert multiple functions to maintain homeostasis of the decidual microenvironment. For instance, dNK cells can modulate trophoblast invasion^8^, promote fetal growth^9^, and regulate immune tolerance^10^. These cells can also exert effector functions upon exposure to exogenous stress^11^.

Decidual macrophages comprise approximately 10–20% of decidual leukocytes during the first trimester of pregnancy and have been reported to function as anti-inflammatory cells with M2-like phenotypes^12–14^. Moreover, crosstalk between decidual macrophages and other cells of the decidual immune microenvironment has been reported to maintain overall immune homeostasis at the maternal–fetal interface^15,16^. Decidual macrophages are known to have many functions similar to those of dNK cells, including remodeling of spiral arteries, trophoblast invasion, promotion of angiogenesis, hindering T cell activation, and mediating canonical responses to antimicrobial infections^16–18^. Decidual T cells also have functional roles in both normal and pathological pregnancies^19^. These maternal leukocytes, together with decidual stromal cells and extravillous trophoblasts (EVTs), interact with each other to form a highly complex immune microenvironment^5,20^.

A number of recent studies have employed single-cell RNA sequencing (scRNA-seq) technology to interrogate the cellular composition and inter-cellular communication events that occur at the maternal–fetal interface^6,21–24^. Building from these seminal studies of global cell types, subsequent focus studies of immune cell subsets have been conducted for normal decidua^25–27^. These foundational studies have defined the composition and distribution of various immune cell types of the immune microenvironment of healthy decidua at high resolution. However, the lack of similar high-resolution data for a dysregulated decidua context—as for example in RPL patients—has limited data-driven hypothesis generation about any immune-related patho-mechanisms underlying failed pregnancies or insights which may more fully elucidate the etiology of recurrent miscarriage.

Here, we profiled the decidual immune cells present at the maternal–fetal interface in RPL patients and healthy controls using scRNA-seq. We found dramatic differential distributions of decidual immune cell subsets in RPL patients. Furthermore, we demonstrated a subset of dNK cells that support embryo growth was reduced due to abnormal NK cell development in disease state revealed by Palantir^28^. We also discovered that macrophage subsets mediated inflammatory T cell chemotaxis in patients. Finally, we constructed a disease-specific interaction network between the major cell types in the decidual immune microenvironment. Our results provide guidance for RPL diagnosis and therapeutics in both cellular and molecular perspectives.

## Results

### An atlas of decidual immune cells in RPL patients

**Fig. 1:**
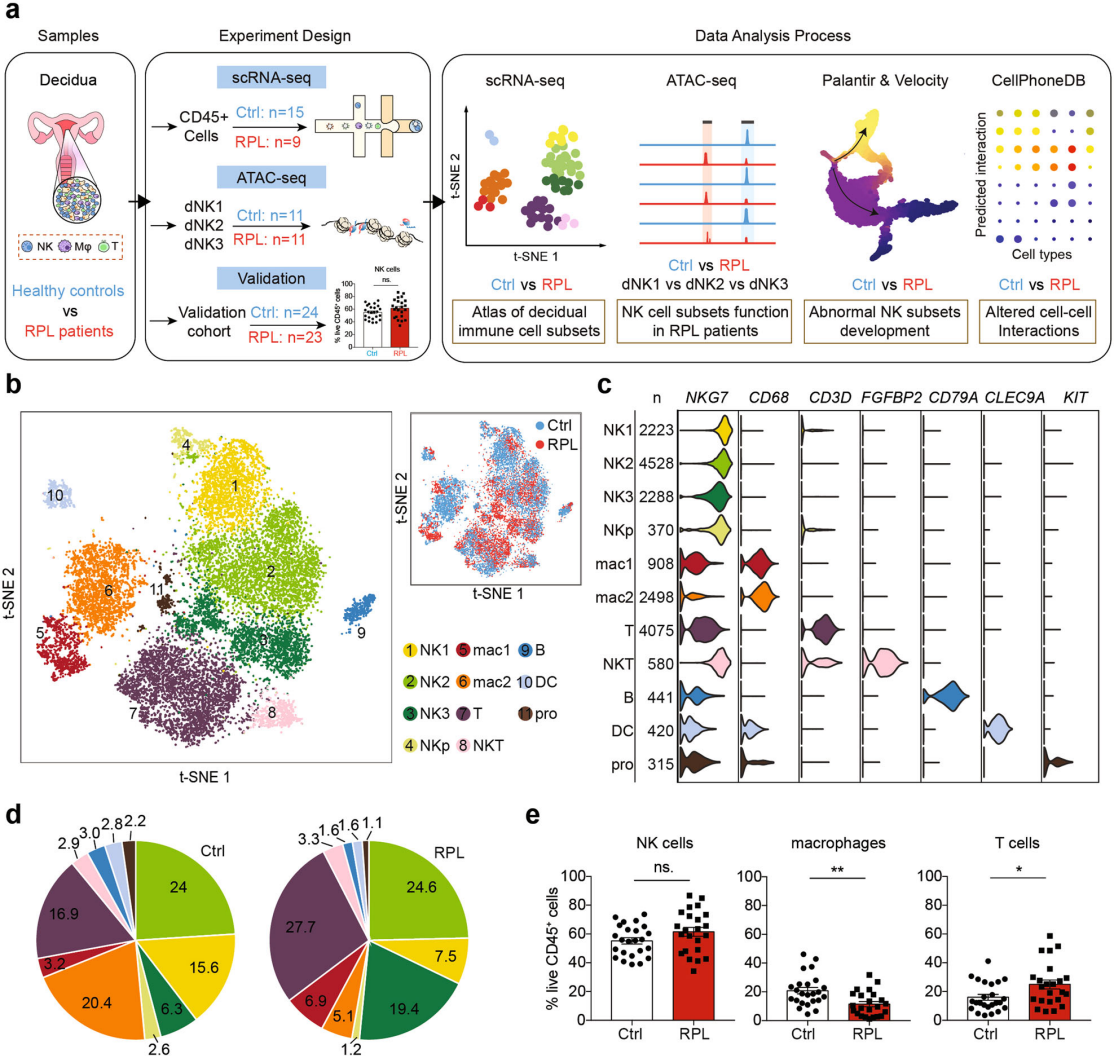
An atlas of decidual immune cells in RPL patients. **a** Flowchart depicting the overall design of the study. Numbers indicate the number of individuals analyzed. **b** A t-SNE projection of the 18,646 total CD45^+^ leukocytes from nine RPL patients and 15 healthy controls, indicating 11 main clusters. Different colors indicate cell clusters and disease status (see legend for key). NKp, proliferating natural killer cells; pro, progenitor cells. **c** Violin plots of selected marker genes (upper row) for multiple immune cell subsets. The first column shows the names of the decidual cell clusters, the second column represents the numbers of cells in each cluster. **d** Pie chart showing the proportion (i.e., % of total sequenced immune cell complement) for each of the 11 cell clusters in healthy controls and RPL patients. **e** Flow cytometry analysis of a larger cohort of healthy controls and RPL patients (including those sequenced in scRNA-seq), showing the proportions of the decidual NK cells, macrophages, and T cells among the gated CD45^+^ leukocytes. ns, *P* > 0.05; *P < 0.05; **P < 0.01; Student’s *t*-test. All points are shown and bars represent means with SEM (*n*_c_ = 24, *n*_p_ = 23; *n*_p_: number of RPL patients, *n*_c_: number of healthy controls).

We obtained 24 human first-trimester decidual samples with normal embryonic karyotypes: 9 from RPL patients and 15 from healthy controls. Specifically, the patients and healthy controls were aged 21–39 years. The mean gestational ages are 7.24 weeks in controls and 8.50 weeks in RPL patients. Four of nine RPL patients experienced three pregnancy loss and five experienced two. The patients were advised to obtain an induced abortion after a diagnosis of pregnancy loss (see Materials and methods, Supplementary Table S1). The decidual sample tissues were digested into single-cell suspensions, and the CD45^+^ leukocytes were then sorted and subjected to scRNA-seq using the 10× platform (Fig. 1a). Low-quality cells were then filtered after rigorous quality control (QC) definition (Supplementary Fig. S1a–d), and we retained a total of 18,646 high-quality CD45^+^ single transcriptomes. Of these, 8504 cells were originated from RPL patients and 10,142 cells from normal controls.

We then applied Seurat^29^ to normalize and cluster the gene expression matrix and identified 11 unique immune cell subsets, which were visualized via *t*-distributed stochastic neighbor embedding (t-SNE) (Fig. 1b). We identified cell lineages, including NK cells, macrophages, T cells, dendritic cells, NKT cells, and immune progenitor cells based on the expression of known marker genes (Fig. 1c). To corroborate our results, we extracted the cells from the healthy controls and compared their transcriptomes with the recently reported scRNA-seq profiles from the decidual immune cells at the maternal–fetal interface^25^ (Supplementary Fig. S2a, b). The two datasets provided extensive overlap in cell identities and the cell-type distributions (Supplementary Fig. S2c, d), suggesting that we have obtained reliable cell atlas of the decidual immune microenvironment in RPL patients and healthy controls.

We also used another integration method, Harmony^30^, to help confirm the reliability of our cell clustering results from Seurat and visualized it with UMAP method (Supplementary Fig. S3a, b). We found strong similarities of the cell clusters processed by Seurat and Harmony (Supplementary Fig. S3c, d), supporting the robustness of our cell clustering results.

Our first general investigation of potential differences between the RPL patients and healthy controls focused on any divergence in the proportion of major immune cell types: there was no apparent difference in the ratio of NK cells, but the RPL patients had decreased macrophage populations and slightly increased T cell populations (Fig. 1d). To validate this result, we measured the distributions of NK, macrophage, and T cells in a larger cohort of RPL patients (*n* = 23) and healthy controls (*n* = 24) using flow cytometry (Supplementary Table S2). As expected, we found a slight but not significant increase in the proportion of NK cells (*P* = 0.0809), a remarkable and significant decrease in macrophages (*P* = 0.0051), and a slight but significant increase in T cells (*P* = 0.0307) (Fig. 1e). Thus, our scRNA-seq provides a useful atlas for identifying disease-associated differences and immune cell population divergence at the maternal–fetal interface during early pregnancy.

### A subset of angiogenic dNK cells are decreased in RPL patients

**Fig. 2:**
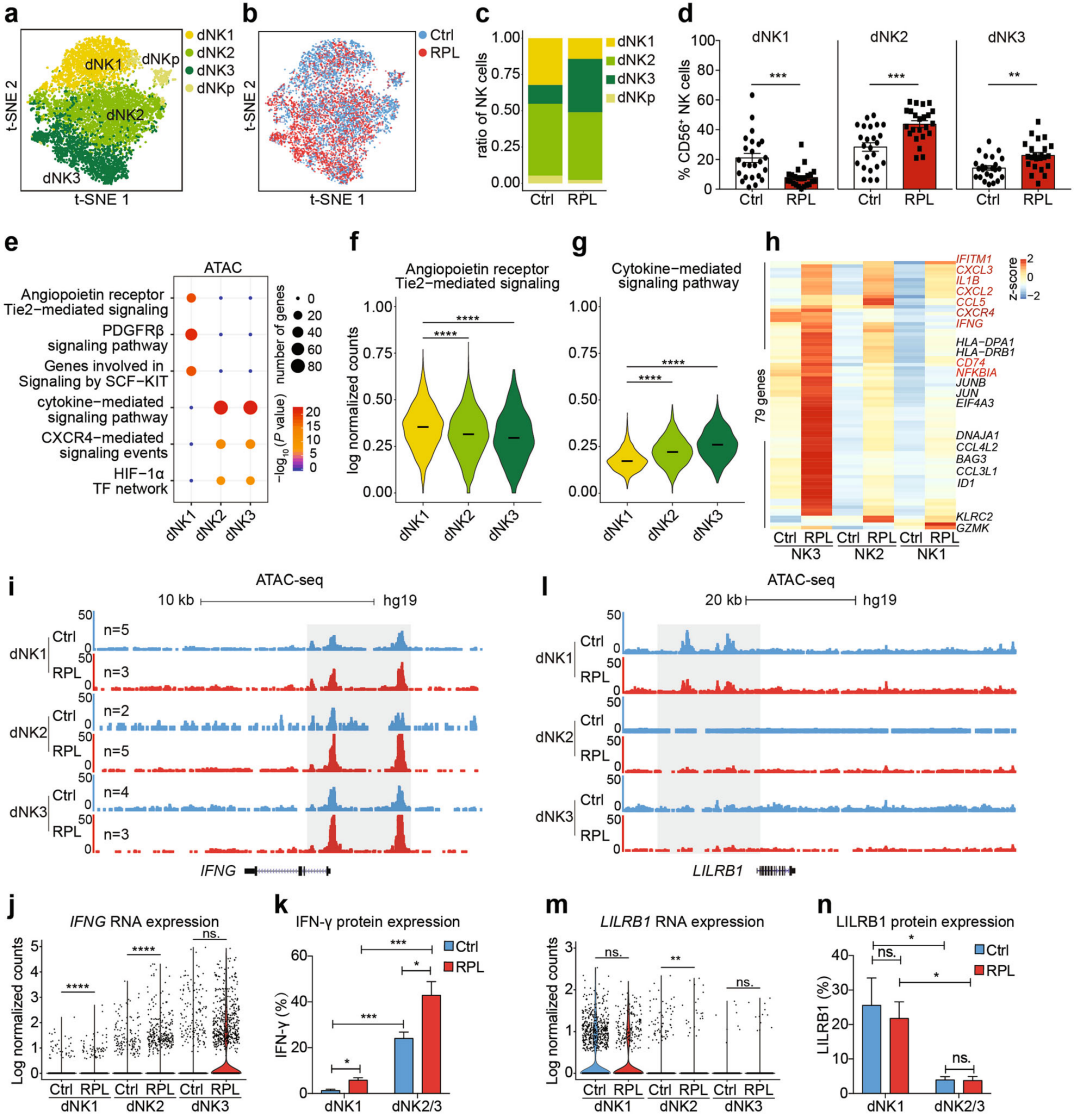
A subset of angiogenic dNK cells are decreased in RPL patients. **a**, **b** t-SNE plots of 9409 dNK cells, indicating four main clusters (**a**) from RPL patients and healthy controls (**b**). Colors indicate cell clusters and disease status. NKp, proliferating natural killer cells. **c** The proportion of dNK1, dNK2, dNK3, and dNKp cell subsets among the 9409 dNK cells from healthy controls and RPL patients. **d** Flow cytometry analysis of a larger cohort of healthy controls (*n*_c_ = 24) and RPL patients (*n*_p_ = 23) to confirm the proportions of dNK1, dNK2, and dNK3 cell subsets among all gated dNK (CD45^+^CD56^+^CD3^−^) cells. **e** Scatter plot of the enriched genomic features in chromatin accessible sites of the three dNK cell subsets by ATAC-seq of the healthy controls (dNK1, *n*_c_ = 5, *n*_p_ = 3; dNK2, *n*_c_ = 2, *n*_p_ = 5; dNK3, *n*_c_ = 4, *n*_p_ = 3). **f**, **g** Violin plots of the expression of genes involved in the signaling pathway “angiopoietin receptor Tie2-mediated signaling pathway” and “cytokine-mediated signaling pathway” in the three dNK cell subsets from healthy controls. **h** Heatmap for unsupervised clustering analysis of the differentially expressed genes between healthy controls and RPL patients, assessed for each of the three dNK cell subsets. Genes colored in red belong to the term of “cytokine-mediated signaling pathway”. **i** UCSC genome browser visualization of the chromatin accessibility profiling at the *IFNG* locus. **j** Violin plots of single-cell RNA expression of the *IFNG* gene in each dNK cell subset in healthy control and RPL patients. **k** Bar graph showing the percentage of IFN-γ expression in dNK1, dNK2, and dNK3 cell subsets from healthy controls (*n*_c_ = 7) and RPL patients (*n*_p_ = 7). **l** UCSC genome browser visualization of the chromatin accessibility profiling at the *LILRB1* locus. **m** Violin plots of single-cell expression of the *LILRB* gene in each dNK cell subset in healthy control and RPL patients. **n** Bar graph showing the percentage of LILRB1 expression in dNK1, dNK2, and dNK3 cell subsets from healthy controls (*n*_c_ = 7) and RPL patients (*n*_p_ = 7). ns, *P* > 0.05; *P < 0.05; **P < 0.01; ***P < 0.001; ****P < 0.0001; Student’s *t*-test. Bars represent means with SEM.

Since dNK cells are the most abundant cell type in the decidual immune microenvironment, we initially explored the dNK cell subsets and their functions at the maternal–fetal interface. We identified three known subsets of dNK cells^25^: CD39^+^ CD18^−^ CD103^−^ (dNK1), CD18^+^ CD103^−^ CD39^−^ (dNK2), and CD18^+^ CD103^+^ CD39^−^ (dNK3), as well as a group of proliferating natural killer cells (dNKp) (Fig. 2a, b; Supplementary Fig. S4a). Whereas our aforementioned analysis indicated no significant differences in the proportion of total dNK cells between RPL patients and healthy controls (Fig. 1d, e), there were obvious differences between patients and controls in the proportions of the dNK cell subsets (Fig. 2c). Specifically, dNK1 cells were significantly decreased in RPL patients, while dNK2 cells were slightly increased and dNK3 cells were significantly increased. We confirmed these findings using flow cytometry analysis of the larger RPL patient cohort (Fig. 2d, *n*_p_ = 23, *n*_c_ = 24, *P* = 0.0004, *P* = 0.0001, *P* = 0.0019, respectively).

To investigate any distinct functions for these three dNK cell subsets, we performed a differential analysis of all the transcriptomes obtained from our scRNA-seq experiment alongside chromatin accessibility profiles from bulk ATAC-seq analysis of sorted dNK subsets. We found that genes of the angiopoietin receptor Tie2-mediated signaling pathway were enriched in the dNK1 cell subset (Fig. 2e): dNK1 cells expressed genes including *CDKN1A*, *RELA*, and *TNIP2* (Supplementary Fig. S4b). Further, we detected elevated expression levels for the 24 genes of this signaling pathway (Fig. 2f). Similar to recently reported findings, we found that dNK1 cells expressed high levels of *KIR* gene family members (encoding the killer immunoglobulin receptor proteins), suggesting that dNK1 cells are likely recognized by EVTs (Supplementary Fig. S4c, d). dNK1 cells also expressed *LILRB1* (Supplementary Fig. S4e, f), which binds to HLA-G proteins expressed on trophoblast cells to increase the secretion of growth-supporting factors^31^.

Regarding the dNK2 and dNK3 cell subsets, these cells had similar extents of chromatin accessibility and had somewhat similar gene expression profiles. Both dNK2 and dNK3 cells were highly enriched for genes of cytokine-mediated signaling pathways (Fig. 2e, g), and the dNK3 cells expressed especially high levels of the immunomodulatory *IFNG* gene (encoding IFN-γ) (Supplementary Fig. S4g, h). These results highlight the functions of the three dNK cell subsets we detected at the maternal–fetal interface, and suggest that the dNK1 cells have embryo growth-supporting activity whereas the dNK2 and dNK3 cells are prone to the cytokine secretion.

Next, seeking etiopathogenic insights about RPL, we examined the functional divergence of the NK cell subsets in RPL patients and healthy controls. Unsupervised clustering of disease-associated differentially expressed genes in the dNK1, dNK2, and dNK3 cell subsets indicated an overall enhancement of cytokine-mediated signaling pathways in the three dNK cell subsets from RPL patients (Fig. 2h; Supplementary Fig. S4i and Table S3). Confirming these findings from our ATAC-seq, scRNA-seq data and flow cytometry analysis showed significantly increased accessibility, expression, and secretion of *IFNG* in RPL patients in all the three dNK cell subsets (Fig. 2i–k), further supporting that dNK cells function to promote an inflammatory environment in RPL decidua. In addition, we found that the expression of *LILRB1* in dNK1 cells was slightly decreased, suggesting that interaction between dNK1 cells and EVTs was weakened under disease conditions (Fig. 2l–n). Collectively, these results indicate that, in RPL decidua, the normal angiogenic function of dNK cells is weakened, and this is accompanied by an enhancement of cells that exert pro-inflammatory dNK functions and an apparent reduction in receptivity for trophoblasts.

### Aberrant differentiation trajectory impairs dNK1 cell subset accumulation in RPL patients

**Fig. 3:**
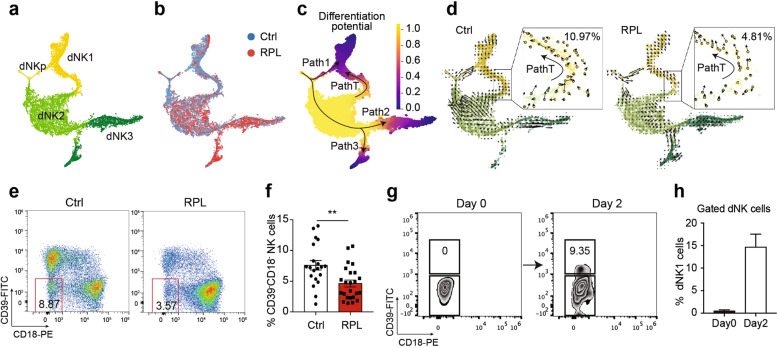
Aberrant developmental trajectory impairs dNK1 cell subset accumulation in RPL patients. **a**–**c** Palantir’s t-SNE map of the total dNKp, dNK1, dNK2, and dNK3 cell subsets (**a**), cells colored by the conditions of RPL patients and healthy controls (**b**), and cells colored by Palantir differentiation potential (**c**). **d** Velocity field projected into the t-SNE map of the dNKp, dNK1, dNK2, and dNK3 cell subsets in the healthy controls (left) and RPL patients (right). Arrows show the local average velocity evaluated on a regular grid. The right frame, velocities of Path T cells shown on the t-SNE map. **e** The proportion of CD39^−^ CD18^−^ dNK cells among gated dNK (CD45^+^CD56^+^CD3^−^) cells from healthy controls (left) and RPL patients (right). **f** Bar plot of the proportion of CD39^−^ CD18^−^ NK cells for multiple samples in healthy controls (*n*_c_ = 20) and RPL patients (*n*_p_ = 24). **g** Percentage of CD39^+^ dNK1 cells before (left) and after (right) CD39^−^CD18^−^ NK cells cultured in vitro for 2 days. **h** Percentage of CD39^+^ dNK1 cells before and after CD39^−^CD18^−^ NK cells cultured in vitro for 2 days. Results in **g** were representative in five independent experiments. **P < 0.01. Significance was evaluated with Student’s *t*-test. All points are shown and bars represent means with SEM.

We then investigated the specific trajectories of the three dNK subsets throughout the course of dNK cell differentiation in the decidua. We applied a high-resolution pseudo-time prediction algorithm Palantir^28^ to construct the differentiation potential trajectory of all dNK cells from RPL patients and healthy controls (Fig. 3a–c and Supplementary Fig. S5a). We found three developmental branches where dNKp differentiate into dNK1 cells (Path 1) and into two distinct branches of dNK2 and dNK3 cells (Path 2 and Path 3) (Fig. 3c and Supplementary Fig. S5a). We also identified a differentiation pathway wherein dNK2-like cells with an apparent tendency to transform into dNK1 cells (Path T) (Fig. 3c). Our discovery of this differentiation pathway illustrates a previously unknown source of pregnancy-promoting dNK1 cells in the decidua.

We next integrated our single-cell data for healthy individuals with the decidua profiles from a previous study^25^ and successfully confirmed that Path 1, Path 2, and Path T also exist in healthy individuals (Supplementary Fig. S5b). In contrast, this integrative analysis indicated that Path 3 is apparently RPL-specific. Further, by comparing the gene expression patterns of cells in Path 2 and Path 3, we noticed that genes related to inflammatory responses were enriched in Path 2 cells, indicated from the expression of *TGFB1*, *NFKB1*, and *REL* (Supplementary Fig. S5c, d). Path 3 cells featured enriched expression for genes of cytokine-mediated signaling pathways, such as *IFNG, TNF* (Supplementary Fig. S5c, e), consistent with the aforementioned increase in cytokine-mediated signaling pathways in the dNK3 cells of RPL patients.

In addition to such pseudo-time analyses, the future state of individual cells can be predicted based on the time derivative of the gene expression state in each cell in terms of RNA velocity^32^. We applied this approach to reveal the differentiation trend of every single cell at one stage to its future stage. As expected, RNA velocity predicted that dNKp cells differentiate and bifurcate into both dNK1 and dNK2 cells (Supplementary Fig. S5f). Interestingly, we found a clear difference in the proportion of Path T cells between RPL patients and healthy individuals, with patients having significantly smaller subsets of Path T cells (Fig. 3d). More specifically, healthy decidua had an average of 540 (540/4923, 10.97%) such cells, whereas RPL patients had an average of only 216 (216/4486, 4.81%) cells, suggesting a weaker tendency of cells transform into dNK1 cells. Note that we successfully confirmed the presence of Path T cells based on Smart-seq2 single-cell transcriptome data for healthy decidua^25^ (Supplementary Fig. S5g).

To characterize these Path T cells in greater detail, we examined the expression of dNK cell subset marker genes in our RNA-seq data and found that Path T cells expressed low levels of *CD39* and *CD18* genes (Supplementary Fig. S5h). A subsequent flow cytometry analysis showed that the number of CD39^−^CD18^−^ dNK cells was significantly decreased in RPL patients compared to healthy controls (Fig. 3e, f, *P* = 0.0026), suggesting that this disease may be affected by a diminished source of dNK1 cells. We then sorted the CD39^−^CD18^−^ dNK cells and cultured them in vitro and confirmed that these cells can indeed successfully transform into dNK1 cells (Fig. 3g, h). Thus, beyond empirically illustrating developmental plasticity for the dNK cell population, our findings suggest that impaired accumulation of the CD39^−^CD18^−^ dNK cells may lead to a decreased number of dNK1 cells, and thereby insufficient support for fetal growth in RPL patients.

### Disease characteristics and cell–cell interactions of macrophage and T cells

**Fig. 4:**
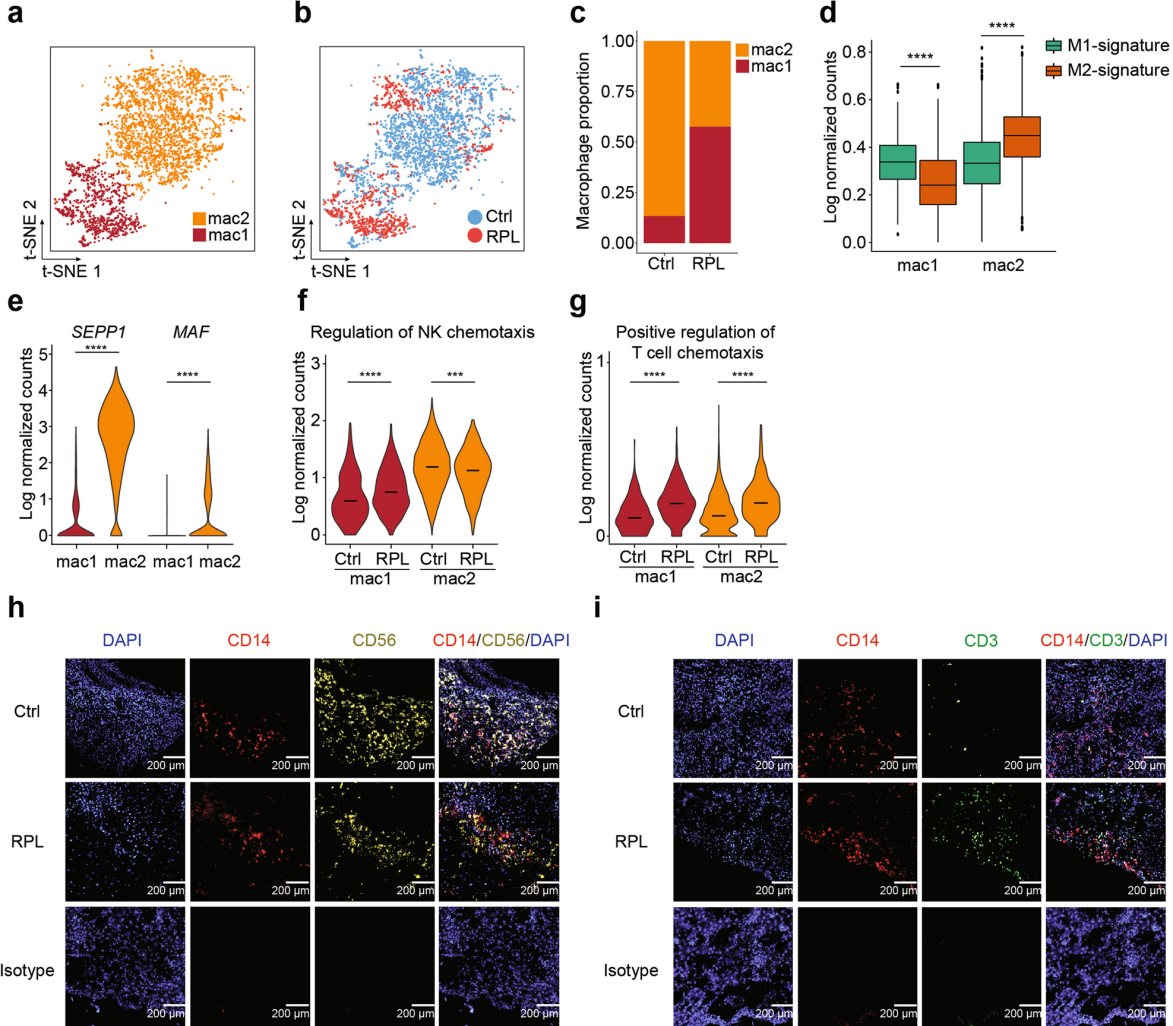
Macrophages enhance cytokine-mediated pathways in RPL patients. **a**, **b** t-SNE plots of macrophages from RPL patients and healthy controls, indicating the mac1 and mac2 subsets (**a**), and the distribution of RPL patients and healthy controls (**b**). Colors indicate cell clusters and disease status. **c** Bar plot showing the proportions of mac1 and mac2 cell subsets in healthy controls and RPL patients. **d** Box plots of the M1 and M2 macrophage signature genes’ expressions in mac1 and mac2 cell subsets. The box represents the second, third quartiles and median, whiskers each extend 1.5 times the interquartile range; dots represent outliers. **e** Violin plot of *SEPP1* and *MAF* gene expression in mac1 and mac2 cells. **f**, **g** Violin plots of the expressions of genes involved in “regulation of NK chemotaxis” (**f**) and “positive regulation of T cell chemotaxis” (**g**) in mac1 and mac2 cell subsets from healthy controls and RPL patients. **h**, **i** Representative example of immunofluorescence staining of DAPI (**h**, **i**, blue), CD14 (**h**, **i**, red), CD56 (**h**, yellow), CD3 (**i**, green), and overlay from a decidual tissue region from healthy controls and RPL patients and from decidual tissue with negative control staining (bottom row). Results in **h**, **i** were representative in three independent experiments. Scale bars, 200 μm.

After dNK cells, macrophages are the most abundant leukocytes^5^, and we examined the cellular heterogeneity of macrophages in the decidual immune microenvironment. We detected two subsets of macrophages from a total of 3406 single-macrophage transcriptomes: mac1 and mac2 (Fig. 4a). Recalling our initial finding that RPL patients had dramatically reduced overall macrophage populations (Fig. 1e), we found that RPL patients had modestly elevated mac1 populations compared to healthy controls and had remarkably decreased mac2 populations (Fig. 4b, c). We then performed a differential analysis of gene expression between mac1 and mac2 subsets to help characterize any functional differences (Supplementary Fig. S6a). Gene Ontology (GO) enrichment of the differentially expressed genes suggested that mac1 cells apparently function in neutrophil-mediated immunity, while mac2 cells may be functionally associated with the regulation of NK cell chemotaxis (Supplementary Fig. S6b). In order to better characterize the phenotype of macrophage subsets, we performed protein interaction network analysis using differentially expressed genes of macrophage subsets by STRING^33^. We found S100A10, S100A6, S100B, and S100A4 genes were overexpressed in mac1 and PLAU, DAB2, EGR1, etc., were enriched in the mac2 subset (Supplementary Fig. S6c).

To assess the M1/M2 polarization potential of these two macrophage subsets, we examined the expression of M1 and M2 genes^34^ in mac1 and mac2 cells. We found that mac1 cells showed M1 polarization characteristics (Fig. 4d); however, mac2 cells were highly enriched with M2 specific genes (Fig. 4d). For example, *SEPP1* and *MAF* (Fig. 4e)—which are important M2-like macrophage marker and regulator^35^—were both expressed in the mac2 cells. These results suggest that mac2 cells may functionally contribute to successful pregnancy by promoting normal M2 macrophage homeostasis in the decidua.

To investigate how the functions of mac1 and mac2 cells may be altered in RPL patients, we conducted a pairwise comparison of the differentially expressed genes of the macrophage subsets between RPL patients and healthy controls. GO analysis indicated that the functions of mac1 cells were enhanced in the disease state (Supplementary Fig. S6d, e). To our surprise, the enrichment analysis predicted that the functions of RPL patient mac2 cells were altered compared to healthy mac2 cells, for example switching from a “regulation of NK cell chemotaxis” predicted function in healthy mac2 cells to “positive regulation of T cell chemotaxis” in RPL patients (Fig. 4f, g and Supplementary Fig. S6f). We also observed the expressions of genes involved in “NK cell chemotaxis” were decreased in mac2 cells in RPL patients, and those with “T cell chemotaxis” were significantly higher expressed in both mac1 and mac2 cells in RPL patients (Fig. 4f, g). Thus, we applied the immunofluorescence assay to characterize the spatial co-localization between macrophages and NK cells and T cells. Our results indicated that macrophages aggregated with NK cells in the normal decidua, whereas under disease condition macrophages co-localized with T cells (Fig. 4h, i).

**Fig. 5:**
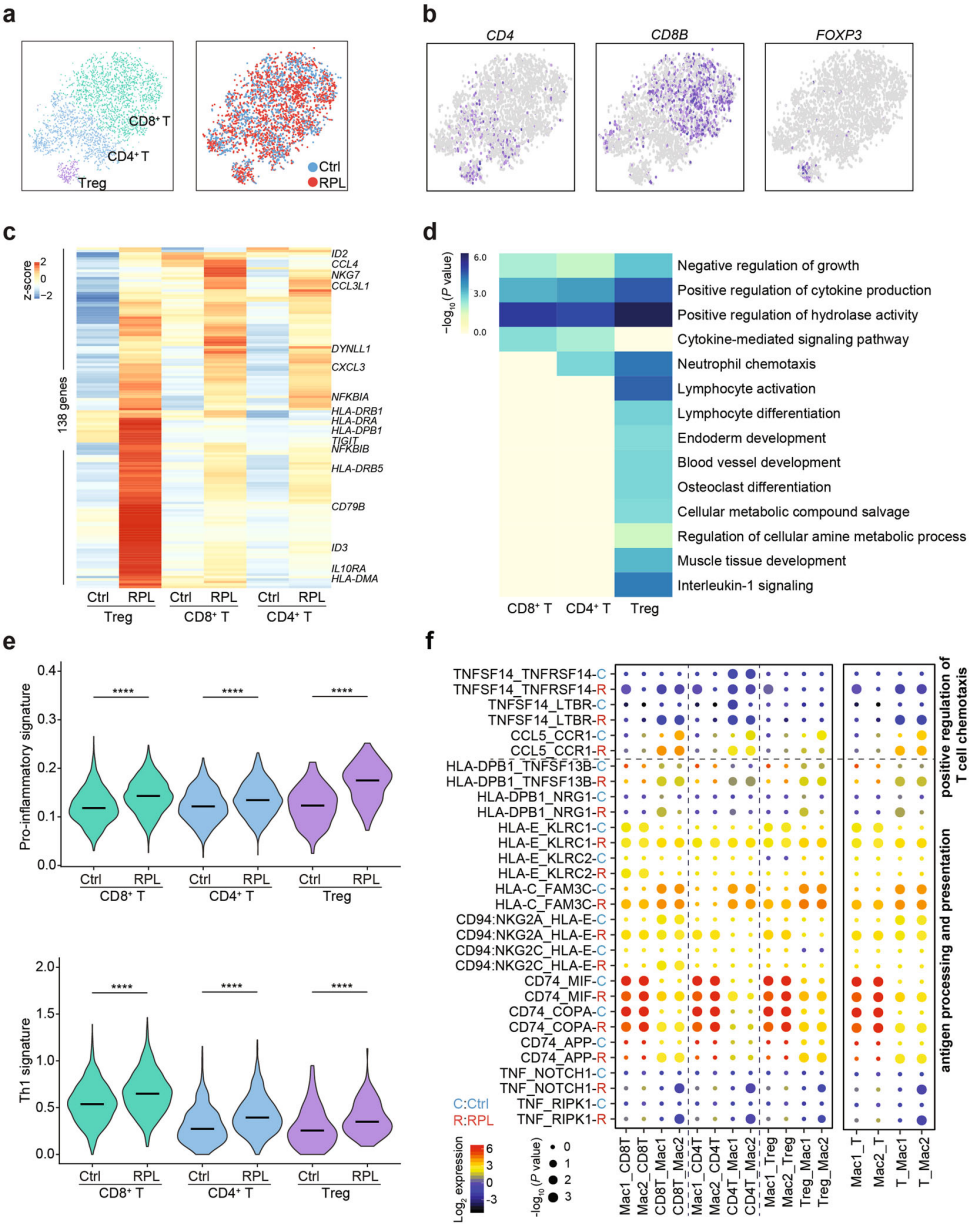
T cell subsets and their interactions with macrophages. **a** t-SNE plots of 4071T cells, indicating three main clusters (left) from RPL patients and healthy controls (right). Colors indicate cell clusters and disease status. **b** t-SNE plots showing the expression of marker genes in each T cell subset. **c** Heatmap for unsupervised clustering of the differentially expressed genes between healthy controls and RPL patients, assessed for each of the three T cell subsets. **d** Heatmap of the enriched genomic features of the differentially expressed genes between healthy controls and RPL patients. **e** Violin plots of the pro-inflammatory and Th1 signature genes’ expressions in T cell subsets from healthy controls and RPL patients. ****P < 0.0001. Significance was evaluated with Student’s *t*-test. **f** Dot plot of the predicted interactions of macrophages with T cells in the RPL patients and healthy controls. *P* values were indicated by circle size. The expression levels of all the interacted genes were indicated by colors, scales on the right.

We next investigated the heterogeneity of T cells from RPL patients and healthy individuals. We re-clustered T cells and identified three T cell subsets including CD4^+^ T, CD8^+^ T, and FOXP3^+^ regulatory T cells (Fig. 5a, b). Unsupervised clustering of disease-associated differentially expressed genes in the CD4^+^ T, CD8^+^ T, and Treg cell subsets indicated an overall enhancement of cytokine-mediated signaling pathways in T cells from RPL patients (Fig. 5c, d). Th1 and pro-inflammatory signatures^36^ were significantly enhanced in T cells and each T subset of RPL patients (Fig. 5e). To further illustrate the molecular basis of the crosstalk between macrophages and T cells, we applied the CellPhoneDB^25^ algorithm to analyze the enrichment of interaction pairs. And we found TNFSF14-TNFRSF14, TNFSF14-LTBR, and CCL5-CCR1 receptor/ligand pairs involved in the “positive regulation of T cell chemotaxis” were enhanced in RPL patients (Fig. 5f).

In summary, our analysis of the second and third most common immune cell types in the decidua revealed that RPL patients exhibit reductions in a homeostasis-promoting subset of macrophages. Instead of dNK cells, macrophages in the RPL disease state are prone to interact with Th1-like T cells.

### Alteration of the decidual immune response in RPL patients

The decidual immune microenvironment is a complex system in which multiple immune cells function via interactions with other cells. In addition to our analyses which considered the RPL-associated changes in specific immune cell types including dNK, macrophages, and T cells, we also used the curated receptor/ligand interaction database CellPhoneDB to identify alterations of molecular interactions between the various immune cell subsets and EVT and stromal cells in the RPL patients (see Materials and methods). We found 959 pairs of interactions between immune cells were increased in RPL patients (Supplementary Table S4). Macrophages engage in extensive interactions with all the other immune cells in patients and in healthy controls and associated with 25.86% (248 pairs) of the overall alteration, suggesting a central role in the regulation of the disease (Supplementary Fig. S7a, b).

**Fig. 6:**
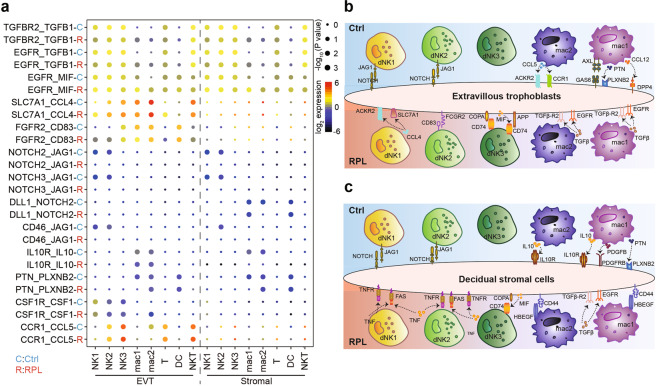
Alteration of decidual immune responses in RPL patients. **a** Dot plot of predicted interactions of immune cells with EVTs and with stromal cells in the RPL patients and healthy controls. *P* values were indicated by circle size. The expression levels of all the interacted genes were indicated by colors, scales on the right. **b**, **c** Summary illustrations depicting the altered interactions of decidual immune cells with EVTs (**b**) and stromal cells (**c**) in healthy controls (up) and RPL patients (down).

To further explore the immune microenvironment of the maternal–fetal interface in RPL patients, we integrated our single-cell decidua data with the scRNA-seq data from healthy EVTs and healthy stromal cells^25^. This dataset integration enabled predictions for the interactions of the eight major immune cell types from RPL patients with EVTs and stromal cells (Fig. 6a). As expected, several receptor/ligand pairs (e.g., CSF1R-CSF1 and CCR1-CCL5) connected the dNK subsets with EVTs in the healthy decidua. In contrast, several other receptor/ligand pairs in immune cell subsets with EVTs and stromal cells were dysregulated according to the RPL decidua data. The strength of interaction through the NOTCH signaling pathway (e.g., NOTCH3-JAG1, NOTCH2-JAG1) between dNK1 and dNK2 cells with EVTs and with stromal cells was significantly reduced in the RPL patients. We summarized the RPL-associated changes in receptor/ligand interactions between immune cells and both the EVTs and stromal cells (Fig. 6b, c and Supplementary Table S5). Collectively, these findings illustrated the molecular basis of cell–cell interactions at the maternal–fetal interface in an inflamed state, leading to a better understanding of the mechanisms of reproductive failure in RPL patients.

## Discussion

The immune microenvironment at the maternal–fetal interface is essential for maintaining normal embryonic development^37^. Previous studies have illustrated the landscape of decidual immune cells and their intercellular interactions at the maternal–fetal interface in healthy people^25^. Here, we present single-cell transcriptomes of the decidual immune microenvironment in patients with RPL. We identified that RPL patients have a remarkably decreased number of a particular subset of dNK cells (dNK1), which may contribute to the secretion of growth-promoting factors to promote normal pregnancy^38^. Meanwhile, RPL patients harbor significantly increased numbers of a pro-inflammatory subset of dNK cells (dNK3) that produce cytokines. We also characterized a population of dNK2-like (Path T) cells which can transform into dNK1 cells, and discovered that the RPL patients have dramatically fewer Path T cells than healthy controls. In addition to dNK cells, we also found that macrophages function differently in normal pregnancy environments and disease states. The former tend to recruit NK cells to maintain immune tolerance, while the latter tend to recruit T cells with an inflammatory signature in RPL patients. Finally, we provide a series of detailed maps presenting the global interactions between our sequenced decidual immune cells and both EVTs and stromal cells, enabling ligand/receptor level hypothesis generation about the likely causes underlying pregnancy failure.

Studies have reported that the normal pregnancy decidual immune microenvironment presents a Th2-biased situation, while Th1-type immunity may lead to pregnancy failure^39^. For one, Th1 cells prevent growth and differentiation of trophoblast through a mechanism that depends on proinflammatory cytokines^40,41^. Indeed, our study found that the overall decidual immune microenvironment of RPL patients presented as primarily Th1-type: the number of dNK2 and dNK3 cell subsets with cytokine-secreting functions were increased in RPL patient decidua, and the proportion of inflammatory macrophage sub-population mac1 was also increased, consistent with recent publication^42^. This mac1 increase was accompanied by a substantial decrease in the mac2 macrophage subset, which was enriched with M2 signature genes and might function to maintain immune homeostasis^43^. However, how RPL macrophage subsets lead to increased cytokine secretion from dNK cells (e.g., IFNG) and promote the chemotaxis of inflammatory T cells still requires further investigation. Viewed collectively, these findings suggest that therapeutically adjusting the proportions of the decidual immune cell subsets may help restore normal pregnancy.

There are inevitable difficulties in obtaining the optimum tissues from patients with RPL who are unlikely to request termination^44^. Although we had taken special care and followed the published procedures^10^ when we collected decidua samples from RPL patients and healthy controls (see Materials and methods), there is a limitation that we obtained samples from women at the time of the pregnancy loss and not at an elective termination in women with this clinical history. Since the decidual samples from RPL patients were collected after fetal demise, it is still possible that some alterations of decidual immune cells we observed in this study could be the consequence of the fetal death. Our empirical data, while not fully satisfactory, delineate the dNK cell plasticity which is associated with the impaired accumulation of pregnancy-promoting dNK1 cells in patients, and improve our understanding of the heterogeneity and interaction of cells at the maternal–fetal interface in disease status, providing clues for future treatment of the disease.

## Materials and methods

### Human samples

Normal decidual samples with no previous pregnancy loss were obtained from elective terminations of apparently normal pregnancies. The elective termination was performed via dilation and curettage (D&C). For the decidua samples from abnormal pregnancies, the patients were advised to obtain an induced abortion after a clinical diagnosis of embryo demise. The diagnosis of embryo demise was following the guidelines from First Affiliated Hospital of University of Science and Technology of China, such as mean sac diameter of 25 mm or greater and no embryo or absence of an embryo with heartbeat 2 weeks or more after a scan that showed a gestational sac without a yolk sac. Fetal heart activity was assessed using Doppler ultrasound at 7–9 weeks of gestation. When abnormal fetal heart activity was observed, the patients were advised to be tested for serum β-human chorionic gonadotropin levels, with additional ultrasounds every other week. D&C was performed within 24 h of ultrasonographic documentation of fetal loss.

When decidua samples were obtained, chorionic villi and blood clots were identified and carefully separated from the maternal decidua. The chorionic villi were then sent for cytogenetic analysis. Normal embryo karyotypes were identified to enable the exclusion of genetic or mechanical causes for embryo demise. We also excluded patients with clinical symptoms of heavy bleeding and cramps prior to induced abortion. The mean gestational ages are 7.24 weeks in controls and 8.50 in RPL patients. All of the decidua samples were collected from the First Affiliated Hospital of the University of Science and Technology of China. Before surgery, informed consent was obtained from each patient. Ethical approvals were obtained from the ethics committee of the University of Science and Technology of China.

### Cell isolation

Fresh decidual tissues were washed extensively in phosphate-buffered saline with 100 IU/mL penicillin/streptomycin and sheared into tiny pieces. Mononuclear lymphocytes were released by digesting the tissues with 1 mg/mL collagenase IV (Sigma) in RPMI 1640 at 37 °C for 1 h, with shaking at 250× r.p.m. The suspensions were filtered via 70 μm nylon mesh cell strainers (i-Quip) and then loaded on a Ficoll-Paque density gradient for separation of decidual cells, which include CD45^+^ immune cells and stromal cells. Next, decidual CD45^+^ cells were sorted and cryopreserved according to the official recommendations from 10× Genomics for scRNA-seq analysis. For ATAC-seq analysis of the dNK cell subsets, dNK1 cells (CD45^+^CD3^−^CD56^+^CD39^+^CD103^−^CD18^−^), dNK2 cells (CD45^+^CD3^−^CD56^+^CD39^−^CD103^−^CD18^+^), and dNK3 cells (CD45^+^CD3^−^CD56^+^CD39^−^CD103^+^CD18^+^) were sorted, and the ATAC-seq libraries were prepared immediately.

### Antibodies and reagents

The following antibodies were used for the analysis of decidual immune cells with FACS, intracellular staining, and cell sorting: PerCP/Cy5.5 anti-human CD45 (HI30), Brilliant Violet^TM^ 421 anti-human CD56 (HCD56), APC/Cy7 anti-human CD3 (SK7), PE anti-human CD14 Antibody (63D3), FITC anti-human CD39 (A1), PE anti-human CD18 (TS1/18), Alexa Fluor® 647 anti-human CD103 (Integrin αE) Antibody (Ber-ACT8), APC anti-human IFN-γ (4S.B3), and APC anti-human CD85j (LILRB1) Antibody (GHI/75). All antibodies above were purchased from BioLegend.

### Flow cytometry

Decidual mononuclear cells were prepared and stained using the aforementioned human mAbs. Homologous IgGs served as negative controls. FACS surface marker staining was performed according to BioLegend antibody instructions. For intracellular staining of cytokines, decidual cells were stimulated with PMA (50 ng/mL, Sigma) and ionomycin (1 μg/mL, Sigma) in the presence of brefeldin (5 μg/mL, BioLegend) for 4 h. After stimulation, the cells were then collected, stained with fluorescein-labeled antibody, washed, and blocked according to the product instructions for BD Cytofix/Cytoperm Cell Permeabilization/Fixation Solution (BD Biosciences).

### ScRNA-seq

We sorted viable CD45^+^ cells from each decidual sample of RPL patients and healthy controls. The cells were then counted and resuspended at a concentration of 1000 cells/μL, aiming for an estimated 8000 cells per library, following the instructions of single-cell 3ʹ solution v2 reagent kit (10× Genomics). Briefly, the cell suspensions were loaded onto a chromium single-cell chip along with reverse transcription master mix and 3ʹ gel beads. After the generation of single-cell gel bead-in-emulsions (GEMs), reverse transcription was performed using a C1000 TouchTM Thermal Cycler (Bio-Rad). The amplified cDNA molecules were then purified with SPRIselect beads (Beckman Coulter). Single-cell libraries were then constructed following fragmentation, end repair, polyA-tailing, adaptor ligation, and size selection according to the manufacturer’s standard protocols. Each sequencing library was generated with a unique sample index. Libraries were sequenced on the Illumina HiSeq X Ten platform.

### Transposome generation

To generate Tn5 transposomes for ATAC-seq library preparation on dNK cell subsets, two oligos (R1, R2) were annealed separately to common pMENTs oligos (5Phos/CTGTCTCTTATACACATCT) at 95 °C for 2 min, with cooling until 14 °C at a rate of 0.1 °C/s. After annealing, the annealed R1 and R2 oligos were mixed at a 1:1 molar ratio, incubated with unloaded transposase Tn5 at 25 °C for 30 min, and then stored at −20 °C for ATAC-seq library construction.

The nucleotide sequences of the two oligos were as follows.

**R1:** TCGTCGGCAGCGTCAGATGTGTATAAGAGACAG.

**R2:** GTCTCGTGGGCTCGGAGATGTGTATAAGAGACAG.

### ATAC-seq library preparation and sequencing

ATAC-seq of dNK cell subsets was performed as previously described^45^, with minor modifications. Briefly, dNK1, dNK2, and dNK3 subsets were sorted using the SH800S sorter (Sony). Samples were obtained from a distinct cohort from those were used for the scRNA-seq. Approximately 50k cells were used per library. Samples were lysed in cold lysis buffer (10 mM Tris-HCl, pH 7.4, 10 mM NaCl, 3 mM MgCl_2_, and 0.1% NP-40 (Roche) for 3 min on ice to prepare the nuclei. Immediately after cell lysis, nuclei were centrifuged at 500× *g* for 5 min and the supernatant was discarded. Nuclei extracts were then incubated with the generated Tn5 transposomes and 5× Tris-DMF tagmentation buffer (pH 8.0, 50 mM Tris-HCl, 25 mM MgCl_2_, 50% DMF) at 37 °C for 30 min. After DNA purification with a MinElute Kit (Qiagen), PCR was performed to amplify the library for 12–15 cycles according to a quantitative PCR reaction for optimum cycles. The PCR thermocycling program was as follows: 98 °C for 30 s; then 98 °C for 10 s, 63 °C for 30 s, and 72 °C for 1 min for the appropriate number of cycles. Following PCR, sample libraries were purified and sequenced using the Illumina HiSeq X Ten platform with the 150-bp paired-end configuration.

### NK cell culturing in vitro

To characterize the “Path T” cells, we sorted CD39^−^CD18^−^ dNK cells, which we cultured in RPMI-1640 with 10% fetal bovine serum. After 2 days, we collected the cultured cells and stained them with fluorescently labeled antibodies as follows: Brilliant Violet^TM^ 421 anti-human CD56 (HCD56), APC/Cy7 anti-human CD3 (SK7), FITC anti-human CD39 (A1), PE anti-human CD18 (TS1/18), and Alexa Fluor® 647 anti-human CD103 (Integrin αE) Antibody (Ber-ACT8). We then performed FACS for the detection of CD39 and CD18 expression in cultured dNK cells.

### Immunofluorescence assay

Decidual tissues from RPL patients and healthy controls were embedded in Optimal Cutting Temperature Compound (O.C.T.) and snap-frozen. The cryostat sections were fixed with 4% PFA and incubated in the blocking buffer at room temperature for 1 h. The fluorescent-labeled antibodies were then added in the dark at 37 °C for 1 h, followed by DAPI staining. PBS + 0.1%BSA was used for washing unlabeled antibodies. The following antibodies were used: FITC anti-human CD3 Antibody (HIT3a, 1:100, Biolegend), PE anti-human CD14 Antibody (63D3, 1:200, Biolegend), and Alexa Fluor® 647 anti-human CD56 Antibody (5.1H11, 1:200, Biolegend). Finally, the decidual tissue sections were evaluated with a confocal microscope (IXplore SpinSR, Olympus). We used Fiji/ImageJ version 2.1.0 to analyze our immunofluorescence images.

### ScRNA-seq data processing

Droplet-based raw data were processed using Cell Ranger (Version 3.0.0)^46^ against the GRCh37 human reference genome with default parameters. First, data from each batch was normalized separately using the NormalizeData function and scaled with the ScaleData function implemented in the Seurat pipeline^29^. Then data from different batches were integrated using the canonical correlation analysis (CCA) method implemented in Seurat^29^. For each subset of immune cells, NK cells and macrophage subtypes were identified based on the clustering analysis of the single-cell profiles from RPL patients and healthy control individuals shown in Fig. 1. T cells were extracted, normalized, re-clustered, and analyzed separately. We retained cells with detected gene numbers between 500 and 3000 and less than 10% mitochondrial UMIs. Moreover, genes expressed in fewer than three cells were also excluded. Downstream data processing and analysis steps, including filtering, normalization, batch removal, dimension reduction, were performed using the Seurat package version 2.3.1. We clustered all the cells based on the integrated gene expression matrix using Seurat with a parameter Resolution=0.6 and generated 11 clusters. In addition, we combined the Droplet-based dataset from Vento-Tormo et al.^25^ with our transcriptome data from healthy controls (1:20 subspaces) using CCA in the Seurat pipeline. To quantify the similarity of the two datasets, we applied matchSCore2^47^ to calculate the Jaccard index of clusters using the top 100 ranking cell-type-specific marker genes. In addition to Seurat, we also used Harmony^30^ to integrate different batches from healthy controls and RPL patients to verify our integration reliability. We used the same gene expression matrix as used in Seurat and performed RunHarmony function in Hamony with default parameters to perform data integration. We then used the same clustering algorithm as that used in Seurat to cluster the cells with resolution 0.3 to generate distinct cell type clusters.

### Differential expression analysis

We performed differential gene expression analysis for our identified cell subsets using the Wilcoxon rank-sum test within the Seurat “FindAllMarkers” command. We also performed differential gene expression analysis between normal individuals and patients using the “FindMarkers” function in the Seurat package. We applied multiple thresholds (fold-change > 1.5, *P* value < 0.01 for NK cells, fold-change > 2, *P* value < 0.01 for macrophage cells) to identify marker genes expressed in each cell subset and differentially expressed genes between normal and patients. We then performed GO analysis using the KEGG pathway and Biological Process database through the GSEApy python package. Protein–protein association network analysis was performed using differentially expressed genes of macrophage subsets by STRING v11^33^ with setting *k*-means clustering method (*k* = 5).

### Single-cell data imputation analysis

We applied SAVER (Version 1.1.1)^48^ to denoise our single-cell transcriptome data with the NK subsets and macrophages. Raw data were processed using the “saver” function with the setting “estimates.only = TRUE”. Finally, we used the imputation data to visualize the differentially expressed genes that were enriched in the RPL patients.

### ATAC-seq data processing

Primary data were processed as described previously^49^. Briefly, we removed adapter sequences and then mapped reads to the hg19 using Bowtie2^50^. The PCR duplicates and reads mapped to chromosome M were removed. The uniquely mapped reads were shifted +4/−5 bp according to the strand of the read. All mapped reads were then extended to 50 bp centered through the cleavage position. Peak calling was performed using MACS2^51^ with the options - f BED -g hs, -q 0.01, --nomodel, and --shift 0. The number of raw reads mapped to each peak at each condition was quantified using the intersectBed function in BedTools^52^. Raw counts in peaks were normalized using the DESeq^53^ package in R. Peak intensity was defined as the log_2_ of the normalized counts. Significance analysis was then performed by pair-wise comparison using DESeq with *P* value < 0.01 and log_2_|fold-change| > 1. GO analysis of cis-regulatory regions was performed with GREAT^54^.

### Developmental trajectory analysis using Palantir

Palantir^28^ is a high-resolution algorithm, which allows computing a continuous probabilistic process to model cell fate choice by applying multiple diffusion components. Here, Palantir was applied to NK subsets in our data and to the integrated dataset. Basically, we used the CCA-aligned subspaces generated from Seurat to replace the low-dimensional principal components subspaces to reduce the batch effects. Twenty diffusion components were selected and computed with default parameters in Palantir. Diffusion components scaled by an Eigen gap were used as inputs and perplexity was set to 200 to generate the t-SNE maps. To accurately define the initial cell state, we imputed a pseudo cell as the start cell by calculating the average gene expression of our identified dNKp cells. A waypoints = 1200 value was applied, and the parameter *k* was set to 50 for datasets.

### Cell velocity analysis

RNA velocity^32^ analysis was performed using the “velocyto run” and “velocyto run_smartseq2” commands (velocyto, version 0.17.17), following this pipeline (https://github.com/velocyto-team/velocyto-notebooks/tree/master/python/DentateGyrus.ipynb) as described. First, cells with the lowest 5 percentile spliced and unspliced counts were filtered out. Genes with less than 50 read counts or were detected in fewer than 20 cells were excluded for spliced molecules and high variation genes set to 3000 were selected. Genes with less than 25 read counts or detected in fewer than 10 cells were also filtered out for unspliced molecules. We also filtered out genes based on a cluster-wise expression with thresholds (unspliced = 0.05, spliced = 0.1). Then, spliced and unspliced molecules counts were normalized separately using the default parameters implemented in the pipeline. To reduce dimensionality, we selected the top 30 principal components to construct a *k*-nearest neighbors algorithm (KNN, *k* = 200) graph, applying the Euclidean distance metric. Finally, velocity-based extrapolation was computed using the assumption of constant velocity under model I. To visualize the predicted results on low-dimensional maps, we projected the velocities onto the t-SNE embedding space generated from Palantir by using the recommended procedures in the pipeline.

### Receptor–ligand interaction analysis

CellPhoneDB^25^ enables analysis of cell–cell communication networks by predicting ligands, receptors, and interactions. To identify potential interactions between NK cells, macrophages, T cells, and other cell types, we applied the CellPhoneDB algorithm to our transcriptome data for normal individuals and patients. Briefly, receptor–ligand interactions were only considered based on the expression of a ligand by one specific cluster and a receptor by another cluster (as least 10% cells expressed). Pairwise comparisons between all cell subsets were performed by randomly permuting cluster labels for all cells 1000 times automatically. And a *P* value for each receptor–ligand in every cluster–cluster interaction was computed using a null distribution. For the EVTs and stromal cells, we finally prioritized interactions with more significant (*P* < 0.05) cell–cell interaction pairs in healthy controls than that in RPL patients, while the selection criteria of interactions between macrophages and T cells was the opposite. We also selected interactions based on biological relevance. Networks were created using Cytoscape (version 3.7.1).

### Statistics

We used unpaired Student’s *t*-tests to assess differences in the proportions of NK cells, macrophage cells, and T cells in healthy controls versus RPL patients. We also used unpaired Student’s *t*-tests to analyze the proportions of dNK cell subsets (dNK1, dNK2, dNK3) and CD39^−^CD18^−^ NK cells (percentage of CD56^+^ NK cells) between the two sample groups. Gene expression and gene set expression between healthy controls and RPL patients were analyzed using unpaired Student’s *t*-tests. To identify marker genes expressed in each subset and the differentially expressed genes between healthy controls and RPL patients, we used the Wilcoxon rank-sum tests implemented in Seurat. A permutation test for the CellphoneDB analysis was used to evaluate the significance of a receptor/ligand pair.

## Supplementary information

Supplementary Information

Supplementary Table S3

Supplementary Table S4

Supplementary Table S5

## Data Availability

Raw data for single-cell RNA-seq and ATAC-seq samples are available in the Genome Sequence Archive (GSA) database as accession number CRA002181. Reviewer access link: https://bigd.big.ac.cn/gsa/s/nBQxzt82.

## Supplementary information

**Supplementary Information** accompanies the paper at (10.1038/s41421-020-00236-z).
