# Supplementary material for: Single-cell profiling of the human decidual immune microenvironment in patients with recurrent pregnancy loss
Source: Cell Discov. 2021 Jan 4;7:1. doi: 10.1038/s41421-020-00236-z (PMC7779601; doi:10.1038/s41421-020-00236-z)
Supplement: Supplementary file 1 — Supplementary Information [file 41421_2020_236_MOESM1_ESM.pdf]

Supplementary information

Supplementary Figures S1-S7

Supplementary Tables S1-S5

a

Batch name	Number of samples that pooled together in each library	Captured cells	Median genes per cell	Mean Reads per cell	Cells passed QC
Batch1	5 Controls	3901	575	94612	1634
Batch2	4 RPLs	3656	1092	108783	2795
Batch3	5 Controls	9117	1359	44463	3691
Batch4	5 RPLs	7357	1147	56062	5709
Batch5	5 Controls	8833	919	43565	4817

b

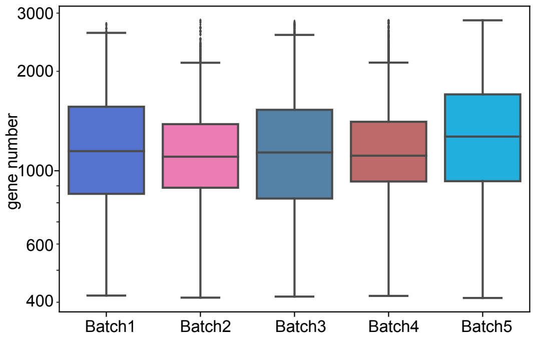

c d

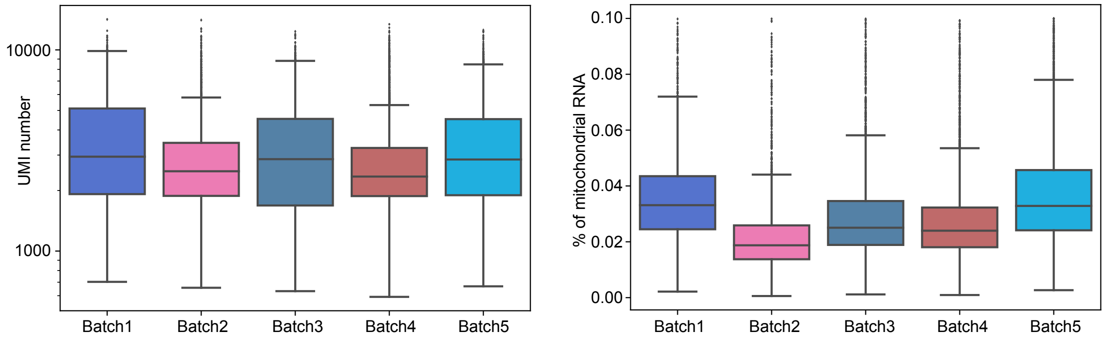

Supplementary Fig. S1. Detailed information and quality control of cells in distinct batches of single-cell data for decidual CD45^{+} leukocytes from RPL patients and healthy control individuals. a, Summary of captured cells, median genes per cell, median UMIs per cell, the number of cells that passed quality control, and the number of samples that were pooled together in each library in distinct batches of single-cell data from RPL patients and healthy control individuals. **b-d,** Box plots showing the distribution of the number of genes (**b**), UMI (**c**) and percentage of mitochondrial RNA **(d)** captured in each batch of single-cell data from RPL patients and healthy control individuals after filtering.

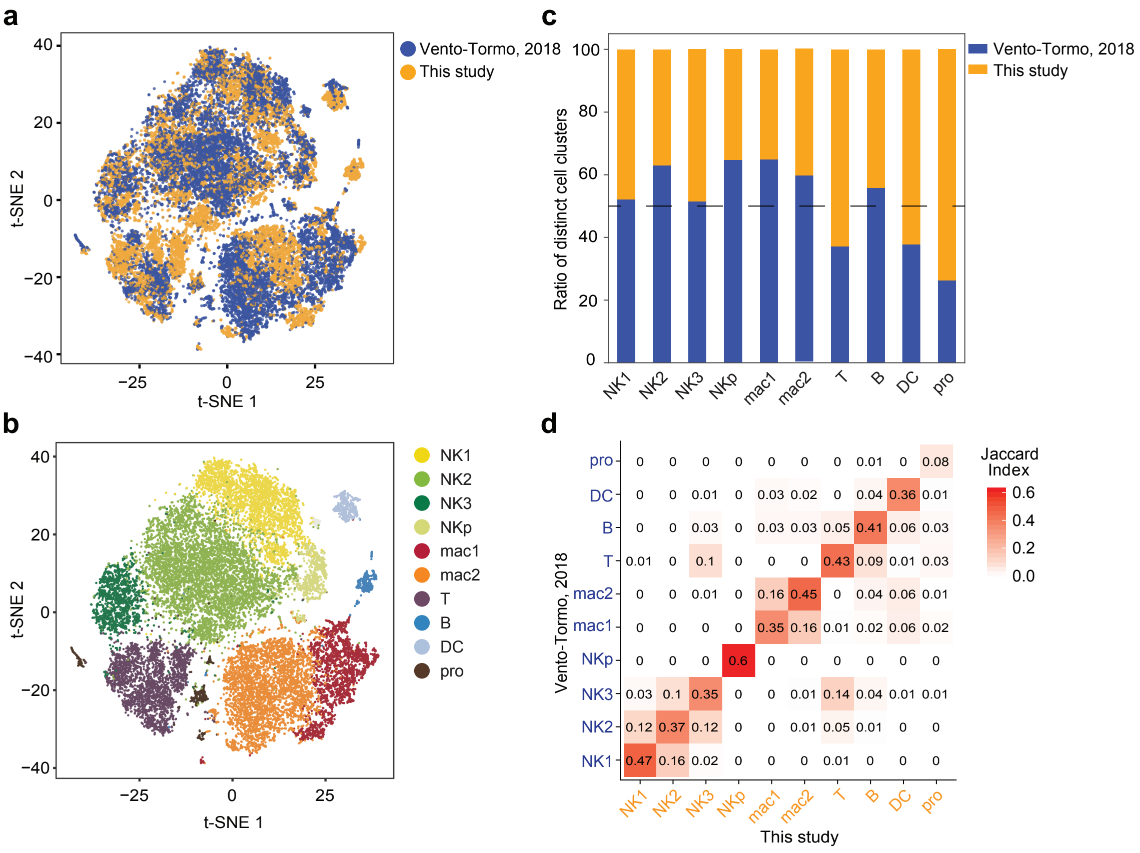

Supplementary Fig. S2. High quality of single-cell datasets. a, **b**, *t*-SNE plots of CD45^{+} decidual cells from our healthy controls and Vento-Tormo et al[1] showing cell origins (**a**) and cell clusters (**b**). Colors indicate cell clusters and origins. NKp, proliferating natural killer cells. **c**, Bar plots of cell ratios of each cell cluster identified in panel **b**. **d**, The Jaccard index matrix of cell clusters identified in panel **b**.

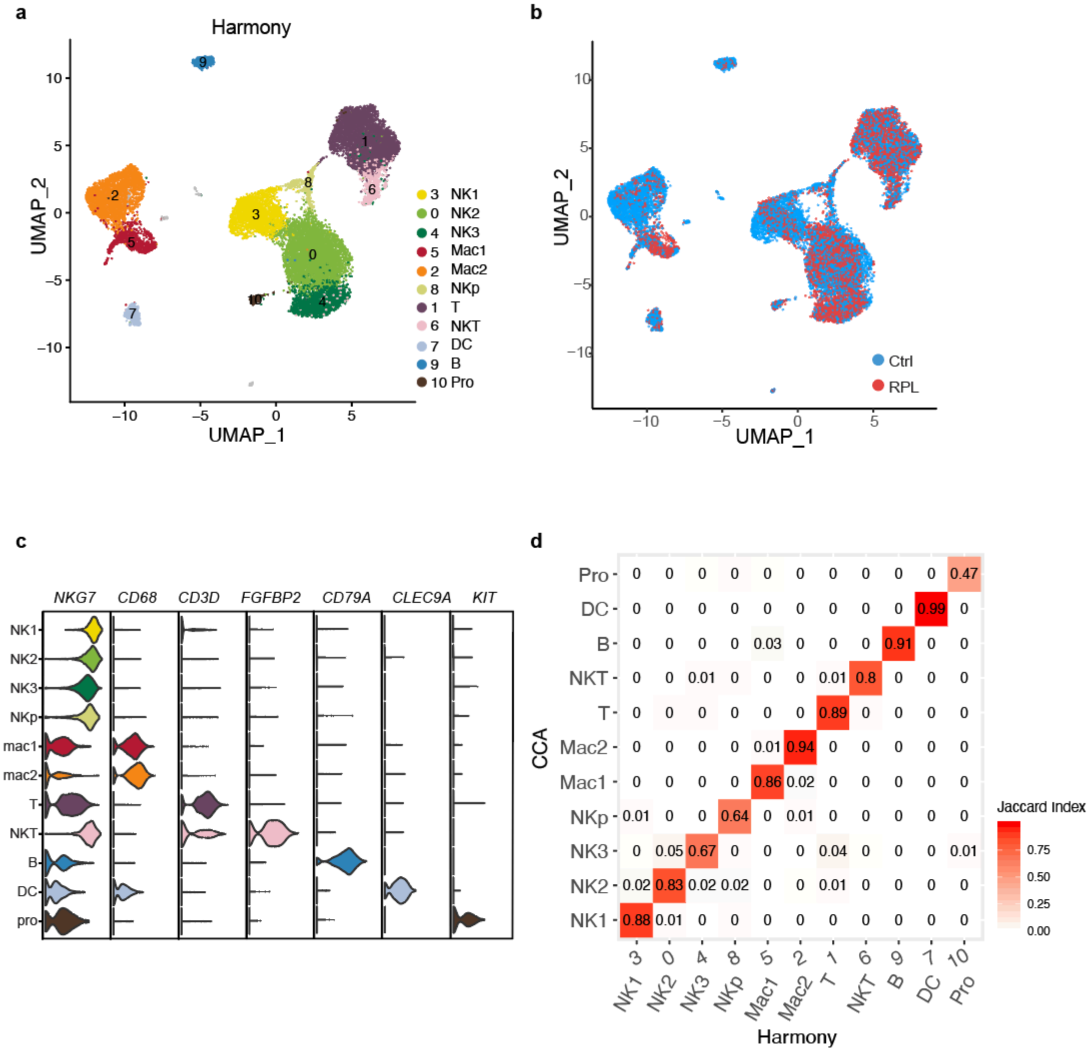

Supplementary Fig. S3. Single-cell profiling of decidual immune cells in RPL patients with healthy controls using Harmony. a-b, The UMAP projection showing single-cell transcriptomes of decidual immune cells in RPL patients with healthy controls integrated by Harmony, colored by clusters (**a**) and status (**b**). **c**, Violin plots of selected marker genes for multiple immune cell subsets. **d**, Jaccard similarities between the cell clusters with integration by CCA methods implemented in Seurat and Harmony.

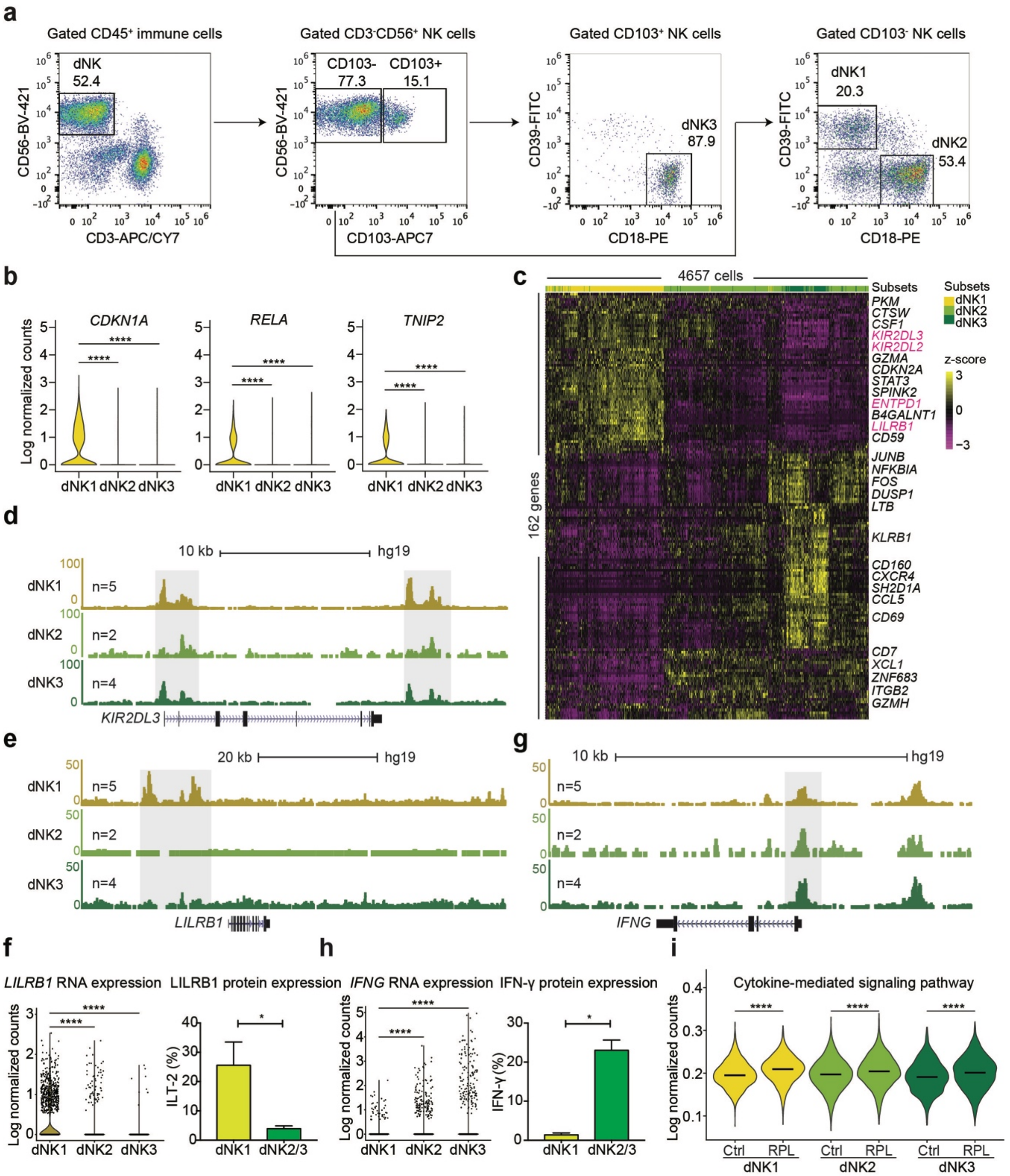

Supplementary Fig. S4. Characteristics of three dNK subsets. a, Gating strategy of dNK subsets in first-trimester decidua. **b,** Violin plots of RNA expression of genes *CDKN1A, RELA, TNIP2* in dNK1, dNK2 and dNK3 cell subsets from healthy controls. **c,** Heat map of unsupervised clustering of total differentially expressed genes between dNK cell subsets from

healthy controls. **d, e,** UCSC genome browser visualization of the chromatin accessibility profiling at the *KIR2DL3* **(d)** and *LILRB1* **(e)** loci in dNK1, dNK2 and dNK3 cell subsets from healthy controls. **f,** Violin plots of single-cell RNA expression of gene *LILRB* (left), and bar graphs showing the expression (flow cytometry based quantification) of protein LILRB1 (right) in dNK1, dNK2, and dNK3 cell subsets from healthy controls. **g,** UCSC genome browser visualization of the chromatin accessibility profiling at the *IFNG* locus in dNK1, dNK2 and dNK3 cell subsets from healthy controls. **h,** Violin plots of RNA expression of gene *IFNG* (left), and bar graphs showing the expression (flow cytometry based quantification) of protein IFN-γ (right) in dNK1, dNK2, and dNK3 cell subsets from healthy controls. **i,** Violin plots of the expressions of genes involved in the signaling pathway "cytokine-mediated signaling pathway" in the three dNK cell subsets from healthy controls and RPL patients. * $P < 0.05$. **** $P < 0.0001$. Significance was evaluated with Student's *t*-test. In panels **f** and **h**, bars represent mean with SEM.

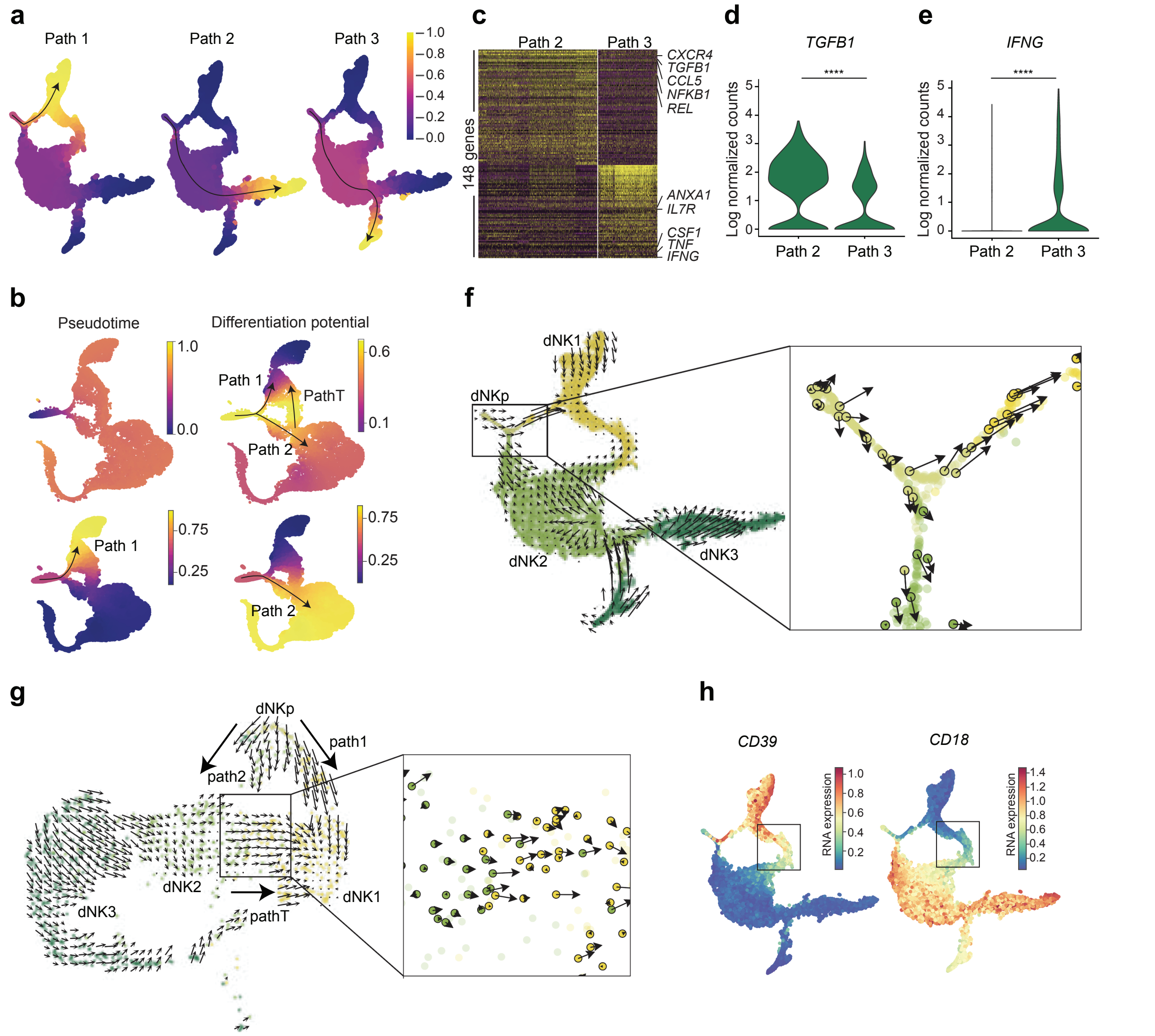

Supplementary Fig. S5. Predicted developmental trajectory of dNK subsets in normal decidua. **a**, Differentiation branches of three Paths predicted by Palantir, colored by differentiation potential. **b**, t-SNE maps of pseudo-time (top, left), differentiation potential (top, right) and differentiation branches (bottom) of dNK cells isolated from our healthy controls and Vento-Tormo et al[1]. **c,** Heat map of differential expressed genes between in Path 2 cells and Path 3 cells. **d, e,** Violin plots of the gene expressions of *TGFB1* **(d)** and *IFNG* **(e)** between Path 2 cells and Path 3 cells. **f,** Velocity field projected into the t-SNE of dNK cells from healthy controls and RPL patients. Right frame, velocities of dNKp cells to dNK1 and dNK2 cells on the

t-SNE map. **g,** Velocity field projected into the t-SNE of dNK cells using Smart-seq2 single cell transcriptomes data from Vento-Tormo et al[1] (n = 817 cells). Right frame, velocities of dNK2 cells to dNK1 cells on the t-SNE map. **h,** t-SNE plots of RNA expression of CD39 and CD18 in 9409 dNK cells. **** $P < 0.0001$. Significance was evaluated with Student's *t*-test.

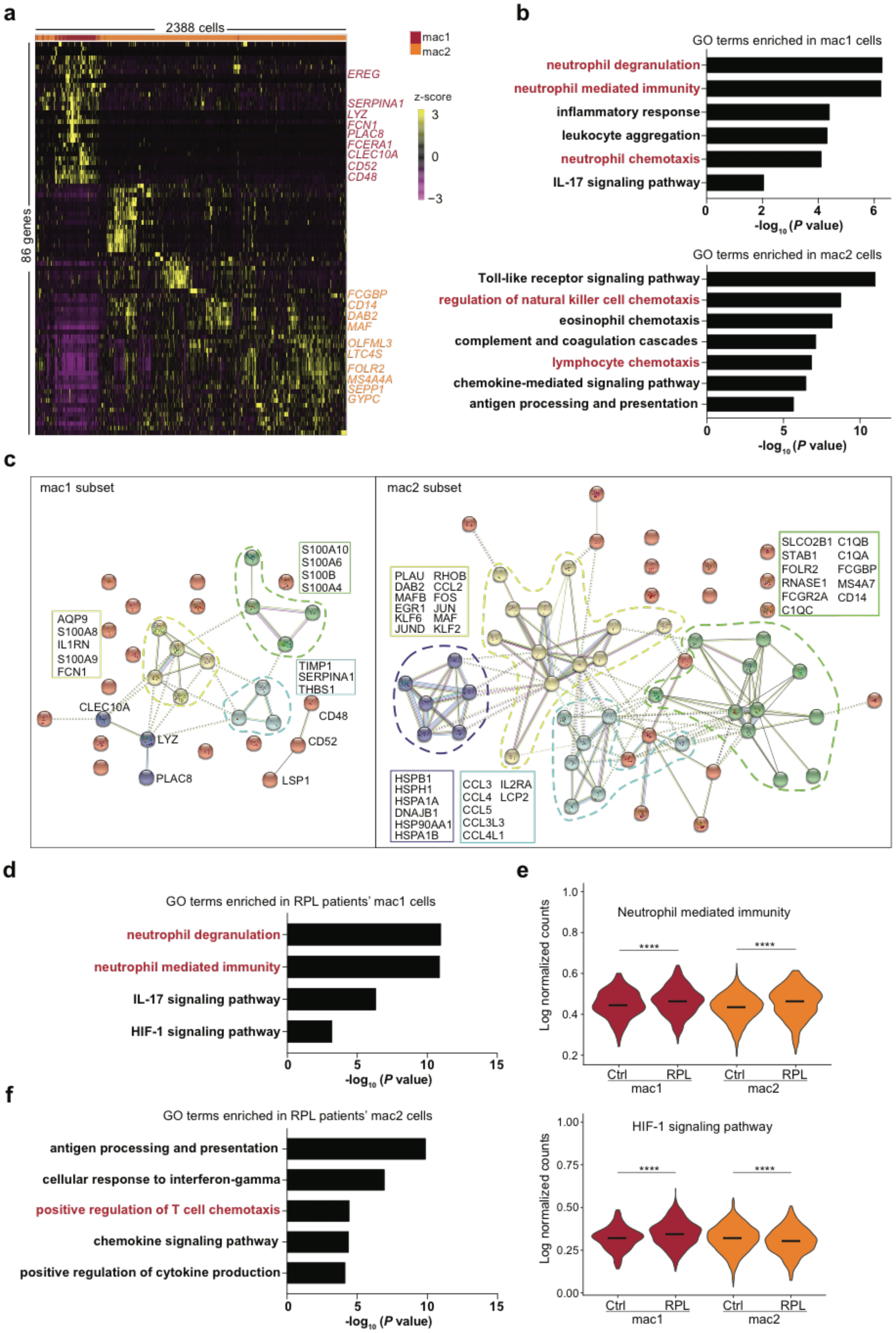
a
2388 cells
mac1
mac2
86 genes
z-score
3
0
−3
EREG
SERPINA1
LYZ
FCN1
PLAC8
FCERA1
CLEC10A
CD52
CD48
FCGBP
CD14
DAB2
MAF
OLFML3
LTC4S
FOLR2
MS4A4A
SEPP1
GYPC
b
GO terms enriched in mac1 cells
neutrophil degranulation
neutrophil mediated immunity
inflammatory response
leukocyte aggregation
neutrophil chemotaxis
IL-17 signaling pathway
-log10 (P value)
GO terms enriched in mac2 cells
Toll-like receptor signaling pathway
regulation of natural killer cell chemotaxis
eosinophil chemotaxis
complement and coagulation cascades
lymphocyte chemotaxis
chemokine-mediated signaling pathway
antigen processing and presentation
-log10 (P value)
c
mac1 subset
S100A10
S100A6
S100B
S100A4
AQP9
S100A8
IL1RN
S100A9
FCN1
TIMP1
SERPINA1
THBS1
CLEC10A
CD48
LYZ
CD52
PLAC8
LSP1
mac2 subset
PLAU RHOB
DAB2 CCL2
MAFB FOS
EGR1 JUN
KLF6 MAF
JUND KLF2
SLCO2B1 C1QB
STAB1 C1QA
FOLR2 FCGBP
RNASE1 MS4A7
FCGR2A CD14
C1QC
HSPB1
HSPH1
HSPA1A
DNAJB1
HSP90AA1
HSPA1B
CCL3 IL2RA
CCL4 LCP2
CCL5
CCL3L3
CCL4L1
d
GO terms enriched in RPL patients' mac1 cells
neutrophil degranulation
neutrophil mediated immunity
IL-17 signaling pathway
HIF-1 signaling pathway
-log10 (P value)
e
Neutrophil mediated immunity
Log normalized counts
****
Ctrl
RPL
mac1
mac2
HIF-1 signaling pathway
f
GO terms enriched in RPL patients' mac2 cells
antigen processing and presentation
cellular response to interferon-gamma
positive regulation of T cell chemotaxis
chemokine signaling pathway
positive regulation of cytokine production
-log10 (P value)

Supplementary Fig. S6. Transcriptomic signatures of mac1 and mac2 subsets. a, Heat map of unsupervised clustering of the differentially expressed genes by comparing mac1 cells with mac2 cells from healthy controls. **b**, Bar plots of GO terms enriched in mac1 cells (top) or mac2 cells (bottom) from healthy controls. **c**, STRING analysis of predicted protein-protein association networks using the differentially expressed genes in mac1 subset (left) and mac2 subset (right). Each color indicates distinct cluster of genes. **d**, Bar plots of GO terms enriched in RPL patients' mac1 cells compare with those from healthy controls. **e**, Violin plots of the RNA expression of genes involved in the pathways of 'neutrophil mediated immunity' (top) and 'HIF-1 signaling pathway' (bottom) in mac1 and mac2 cells from healthy controls and RPL patients. **f**, Bar plots of GO terms enriched in RPL patients' mac2 cells compare with those from healthy controls. **** $P < 0.0001$. Significance was evaluated with Student's *t*-test.

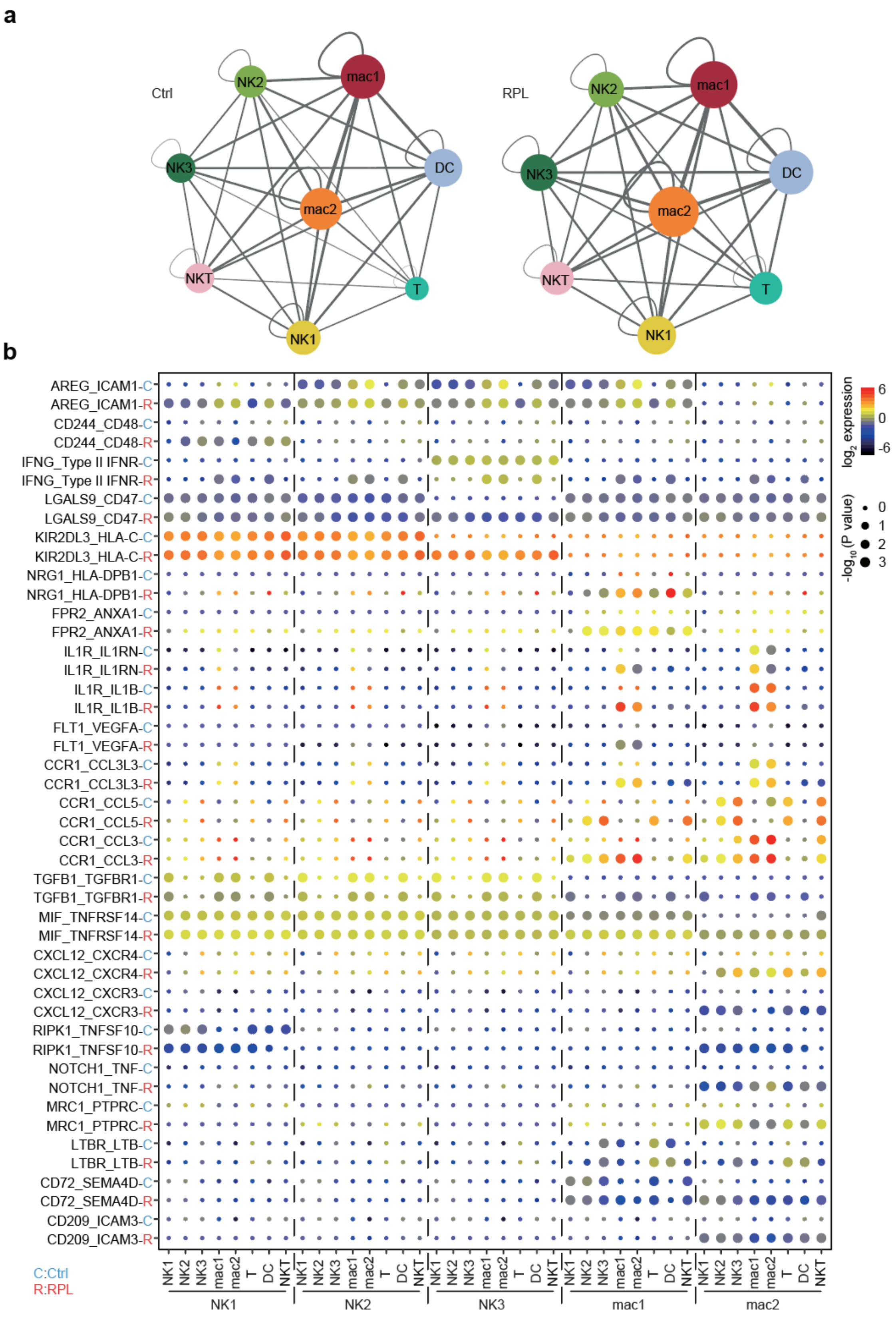
a
Ctrl
RPL
NK1
NK2
NK3
mac1
mac2
DC
T
NKT
b
AREG_ICAM1-C
AREG_ICAM1-R
CD244_CD48-C
CD244_CD48-R
IFNG_Type II IFNR-C
IFNG_Type II IFNR-R
LGALS9_CD47-C
LGALS9_CD47-R
KIR2DL3_HLA-C-C
KIR2DL3_HLA-C-R
NRG1_HLA-DPB1-C
NRG1_HLA-DPB1-R
FPR2_ANXA1-C
FPR2_ANXA1-R
IL1R_IL1RN-C
IL1R_IL1RN-R
IL1R_IL1B-C
IL1R_IL1B-R
FLT1_VEGFA-C
FLT1_VEGFA-R
CCR1_CCL3L3-C
CCR1_CCL3L3-R
CCR1_CCL5-C
CCR1_CCL5-R
CCR1_CCL3-C
CCR1_CCL3-R
TGFB1_TGFBR1-C
TGFB1_TGFBR1-R
MIF_TNFRSF14-C
MIF_TNFRSF14-R
CXCL12_CXCR4-C
CXCL12_CXCR4-R
CXCL12_CXCR3-C
CXCL12_CXCR3-R
RIPK1_TNFSF10-C
RIPK1_TNFSF10-R
NOTCH1_TNF-C
NOTCH1_TNF-R
MRC1_PTPRC-C
MRC1_PTPRC-R
LTBR_LTB-C
LTBR_LTB-R
CD72_SEMA4D-C
CD72_SEMA4D-R
CD209_ICAM3-C
CD209_ICAM3-R
$\log_2$ expression
6
0
-6
$-\log_{10}$ (P value)
0
1
2
3
C:Ctrl
R:RPL
NK1 NK2 NK3 mac1 mac2 T DC NKT
NK1
NK2
NK3
mac1
mac2

Supplementary Fig. S7. Alteration of decidual interactions in RPL patients. a, Receptor/ligand interaction networks depicting cell types as nodes and interactions as edges from healthy controls (left) and RPL patients (right). The size of the circle is proportional to the total number of interactions detected for each cell type; edge thickness is proportional to the absolute number of interactions detected between the connected cell types. **b**, Dot plot of predicted receptor/ligand interactions of immune cells with NK cell subsets (NK1, NK2, NK3) and macrophages (mac1, mac2) in the RPL patients and healthy controls. *P* values were indicated by circle size. The expression levels of all the interacted genes were indicated by color, scales on the right.

Supplementary Table S1. Clinical Data for RPL patients and healthy controls (Single-cell RNA-seq).

	**Age (year)** (Mean±SD)	**Number of pregnancies** (Mean±SD)	**Number of pregnancy loss** (Mean±SD)	**Pregnancy (weeks)** (Mean±SD)
**Controls** (N=15)	28.50 ± 4.86	1.40 ± 0.49	0.0 ± 0.0	7.39 ± 0.83
**RPL** (N=9)	32.40 ± 4.32	2.20 ± 0.40	2.38 ± 0.48	9.03 ± 1.00

Supplementary Table S2. Clinical Data for RPL patients and healthy controls (Validation cohort).

	**Age (year)** (Mean±SD)	**Number of pregnancies** (Mean±SD)	**Number of pregnancy loss** (Mean±SD)	**Pregnancy (weeks)** (Mean±SD)
**Controls** (N=24)	28.45 ± 4.65	1.43 ± 0.49	0.0 ± 0.0	7.01 ± 0.99
**RPL** (N=23)	30.84 ± 4.53	2.35 ± 0.48	2.16 ± 0.49	9.08 ± 1.60

Supplementary Table S3. RPL disease-associated differentially expressed genes in the dNK1, dNK2 and dNK3 cell subsets.

Supplementary Table S4. Interactions of nine immune cells with immune cells in the RPL patients and healthy controls.

Supplementary Table S5. Interactions of nine immune cells with EVTs and with stromal cells in the RPL patients and healthy controls.

References:

1. Vento-Tormo, R. *et al.* Single-cell reconstruction of the early maternal-fetal interface in humans. *Nature* **563**, 347-353 (2018).
